# New continental record and new species of *Austromerope* (Mecoptera, Meropeidae) from Brazil

**DOI:** 10.3897/zookeys.269.4255

**Published:** 2013-02-15

**Authors:** Renato Jose Pires Machado, Ricardo Kawada, José Albertino Rafael

**Affiliations:** 1 Texas A & M University, Department of Entomology College Station, TX, USA; 2 Universidade Federal do Espírito Santo, Laboratório de Entomologia Sistemática, Vitória, ES, Brazil; 3 Instituto Nacional de Pesquisas da Amazonia, Coordenação de Pesquisas em Entomologia, Manaus, AM, Brazil

**Keywords:** Earwigflies, Merope, Neotropical, scorpionflies

## Abstract

A new species of Meropeidae (Mecoptera) from Brazil, *Austromerope brasiliensis* **sp. n.**, is described, representing only the 3rd extant species described in this family and the 1st record of the family from the Neotropical region. The distribution and biogeography of the family are discussed and we propose that Meropeidae originated before continental drift and then divided into two branches, northern and southern, with the breakup of Pangea. Identification keys for the Neotropical families of Mecoptera and for the species of Meropeidae are provided.

## Introduction

Meropeidae is one of the smallest and least known families of Mecoptera. Until now, only 2 extant species were known, *Merope tuber* Newman, 1838 from eastern North America, and *Austromerope poultoni* Killington, 1933 from southwestern Australia (Byers 1973; Dunford et al. 2007). In addition to these 2 species, there is 1 fossil species, *Boreomerope antiqua* Novokschonov, 1995 from the Middle Jurassic, in Siberia (Dunford et al. 2007). These insects are very different from more common mecopterans, e.g., Panorpidae and Bittacidae, since their wings are broad, with elaborate venation, and folded over the abdomen; the body is flattened and the head is opisthognathous, almost cockroach-like in appearance (Penny 1975). They are usually known as earwigflies, because the males have a large genital forceps that resembles the cerci of earwigs (Dermaptera) (Somma and Dunford 2007).

Little is known about the biology of Meropeidae. The adults, which are nocturnal, seem to live on the ground, are capable of stridulation (Sanborne 1982), and generally are collected in Malaise (Dunford et al. 2007; Barrows and Flint 2009) and pitfall traps (Abbott et al. 2007). Immature stages are still unknown (Johnson 1995). The placement of the family within Mecoptera, however, remains under discussion. Meropeidae is very often associated with Eomeropidae because of the similarities between their body and wing shape. Due to their differences from other mecopterans, these 2 families were once classified in the suborder Protomecoptera (Whiting 2002). Earlier, Penny (1975) suggested that Meropeidae was sister to all other families of Mecoptera. The monophyly of the Mecoptera is another open question; some phylogenetic studies support monophyly of the order (Trautwein et al. 2012), others have suggested that Mecoptera is paraphyletic and includes Siphonaptera (Whiting 2002; Beutel and Baum 2008), or that Meropeidae is the sister family only to the most derived families of Mecoptera (Whiting 2002; Grimaldi and Engel 2005). Taking a different approach, the phylogenetic work of Friedrich and Beutel (2010), which was based on thorax morphology, proposed Meropeidae as the sister family of Antliophora (Siphonaptera + Mecoptera + Diptera). Although its phylogenetic placement remains equivocal, in this paper we describe the 3rd extant species in the family based on 1 male specimen recently collected in Brazil. We also provide keys for the Neotropical families of Mecoptera and the extant world species of Meropeidae.

## Material and methods

The specimen described in this work was collected in Rancho Sonho Meu, in the southeastern Brazilian state of Espírito Santo, Domingos Martins municipality. In addition, specimens of *Merope tuber* were examined in this study. Specimens were transferred from 70 to 98.5% ethanol through an ethanol dehydration series and critical point dried using a BAL-TEC 030 critical point drying apparatus. The left wings of *Merope tuber* were removed and attached with glue to a triangular paper card and placed on the same pin with the rest of the body. A Leica M205C stereomicroscope with an attached magnifying lens and Leica DFC 295 video camera were used to examine and photograph specimens. Leica Application Suite V3.6.0 installed on a desktop computer (Windows 7 Professional, Intel Xeon) was used to combine images. Images were subsequently edited in Adobe Photoshop® using various adjustments (e.g., levels, shadows/highlights), tools (e.g., healing brush, clone stamp) and filters (e.g., unsharp mask). Photographs were assembled into plates using Adobe Illustrator®. The specimens of *Merope tuber* and the new species are deposited in the collection of Universidade Federal do Espírito Santo (UFES). The identification key for Neotropical families of Mecoptera was adapted from Machado et al. (2009). Wing terminology follows that proposed by Willmann (1981).

## Results

### Key to the Neotropical families of Mecoptera

**Table:** 

1	Wings large, semi-elliptical, with more than 50 crossveins	2
1’	Wings long, elongated, with less than 30 crossveins	3
2	Forewing Cu1 forked; ocelli present; legs spinose	Eomeropidae
2’	Forewing Cu1 not forked; ocelli absent; legs not spinose	Meropeidae
3	Legs raptorial, tarsus with 1 apical claw	Bittacidae
3’	Legs unmodified, tarsus with 2 apical claws	4
4	Forewing with less than 15 crossveins; Rs with 3 branches. Argentina and Chile	Nannochoristidae
4’	Forewing with more than 20 crossveins; Rs with 4 branches. North America	Panorpidae*

[*Panorpidae distribution is basically Nearctic, but recent records from Southern Mexico (Bicha 2006) required the inclusion of this family in the key.]

### Key to males of world species of Meropeidae

**Table:** 

1	Antennal flagellomeres 2.0× wider than long (Figs 3e, 5a); wing membrane fuscous; costal crossveins slightly parallel to Costa (Figs 2b, 5b); Rs with 10 or more branches (Figs 2b, 5b), tarsal claws with small teeth (Fig. 3f); abdominal tergite IX longer than tergite VIII in dorsal view (Fig. 3b); terminalia with basal segment of forceps subparallel (Fig. 1, 3c)	2
1’	Antennal flagellomeres almost as wide as long (Fig. 7b); wing membrane hyaline; costal crossveins not parallel to Costa (Fig. 9); Rs with 5 branches (Fig. 9); tarsal claws without teeth; abdominal tergite IX as long as tergite VIII in dorsal view (Fig. 7c); terminalia with basal segment of forceps divergent (Figs 7a, 8b). USA	*Merope tuber* (Fig. 6a, lateral habitus)
2	Terminalia with basal segment of forceps with truncated expansion apically (Fig. 3d); forewing Cu1 not connected with M (Fig. 2b). Brazil	*Austromerope brasiliensis* sp. n. (Fig. 2a, lateral habitus)
2’	Terminalia with basal segment of forceps with large spine apically (Fig. 5a); forewing Cu1 connected with M by short distance basally (Fig. 5b). Australia	*Austromerope poultoni* (Fig. 5a, ventral habitus)

#### Austromerope brasiliensis sp. n.

urn:lsid:zoobank.org:act:452F3F6F-BE98-4352-82AC-AE2681DE7D0F

http://species-id.net/wiki/Austromerope_brasiliensis

##### Type material.

Holotype, BRAZIL: Espírito Santo: Domingos Martins: Pico Eldorado, 20°22'27.19"S, 40°39'33.35"W, 05-12.vii.2003, Malaise trap, R. Kawada col. - 1 male (UFES). Condition is good, but with left antennae broken and apex of right hind leg missing.

##### Diagnosis.

**Figure 1.**
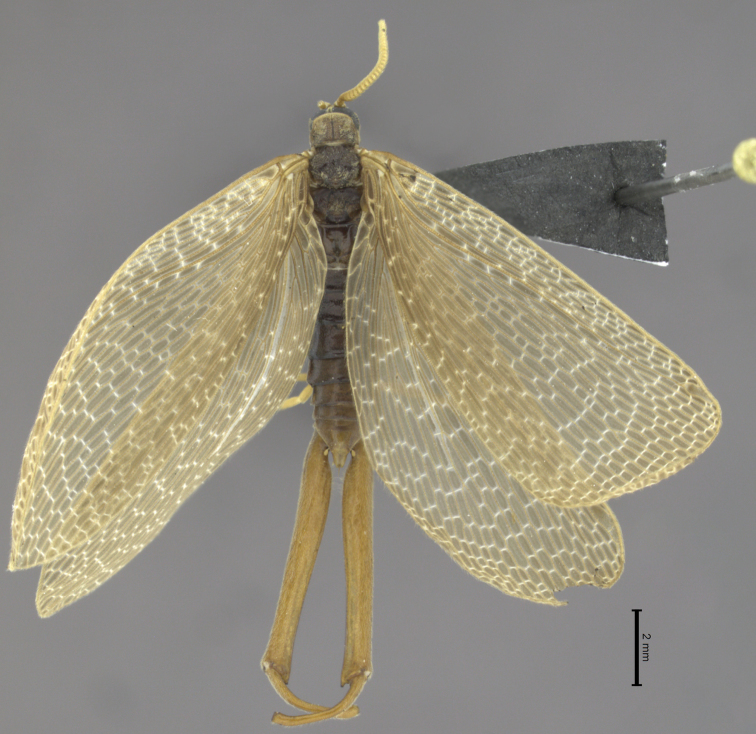
*Austromerope brasiliensis* sp. n. dorsal view.

**Figure 2.**
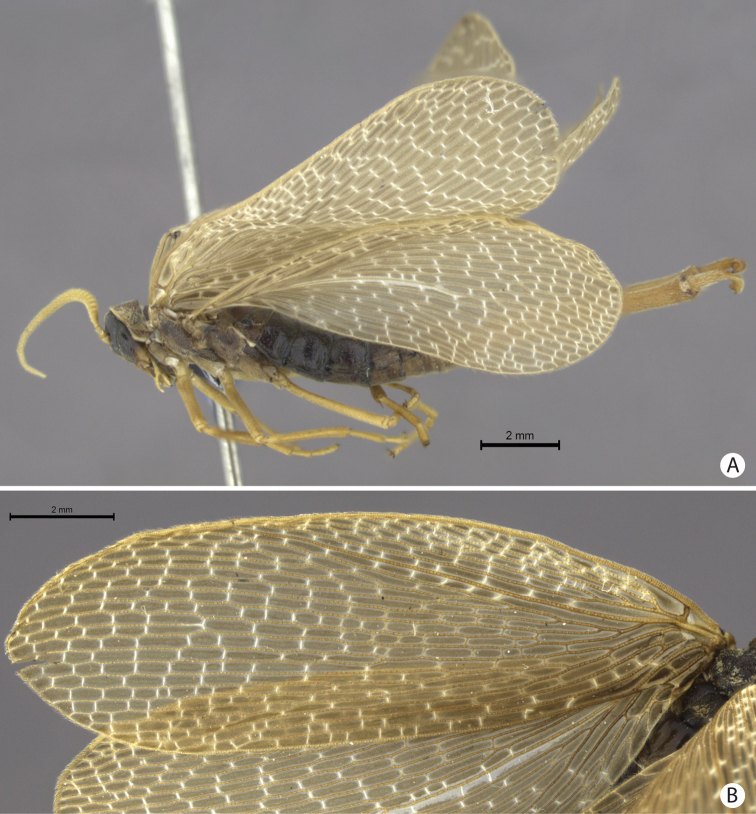
**A–B** *Austromerope brasiliensis* sp. n. **A** lateral view **B** Left forewing.

**Figure 3.**
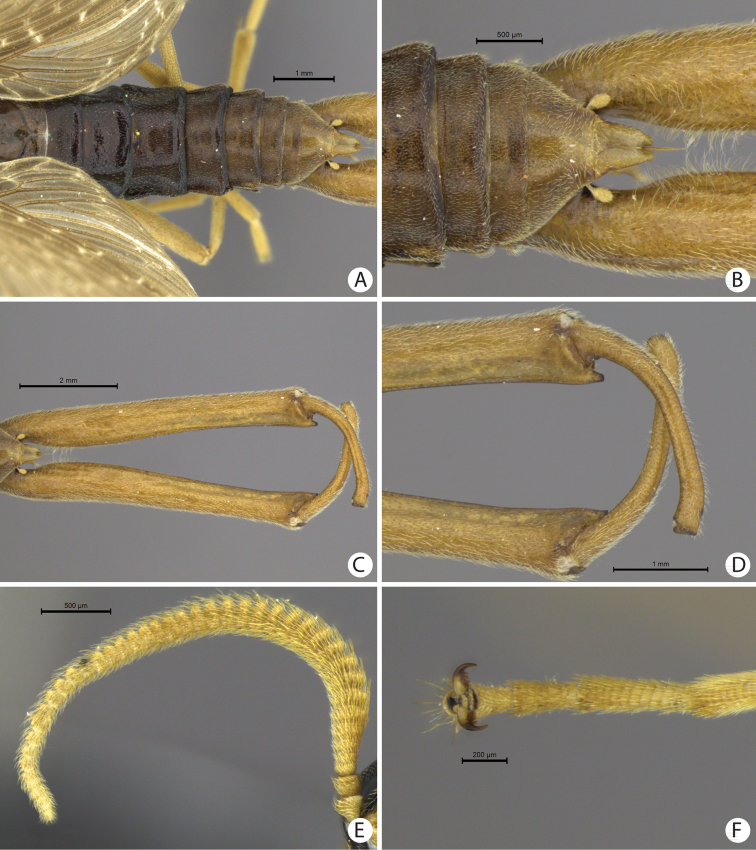
**A–F** *Austromerope brasiliensis* sp. n. **A** Abdomen dorsal view **B** Abdomen tip, dorsal view **C** Terminalia, dorsal view **D** Terminalia tip, dorsal view **E** Antennae, lateral view **F** Pretarsal claw.

This species is characterized by the semi-elliptical wings with many crossveins (Fig. 2b), the large genital forceps (Fig. 1), and by the truncate expansion of the apex of the basal segment of the forceps (Fig. 3d).

##### Description

**Figure 4.**
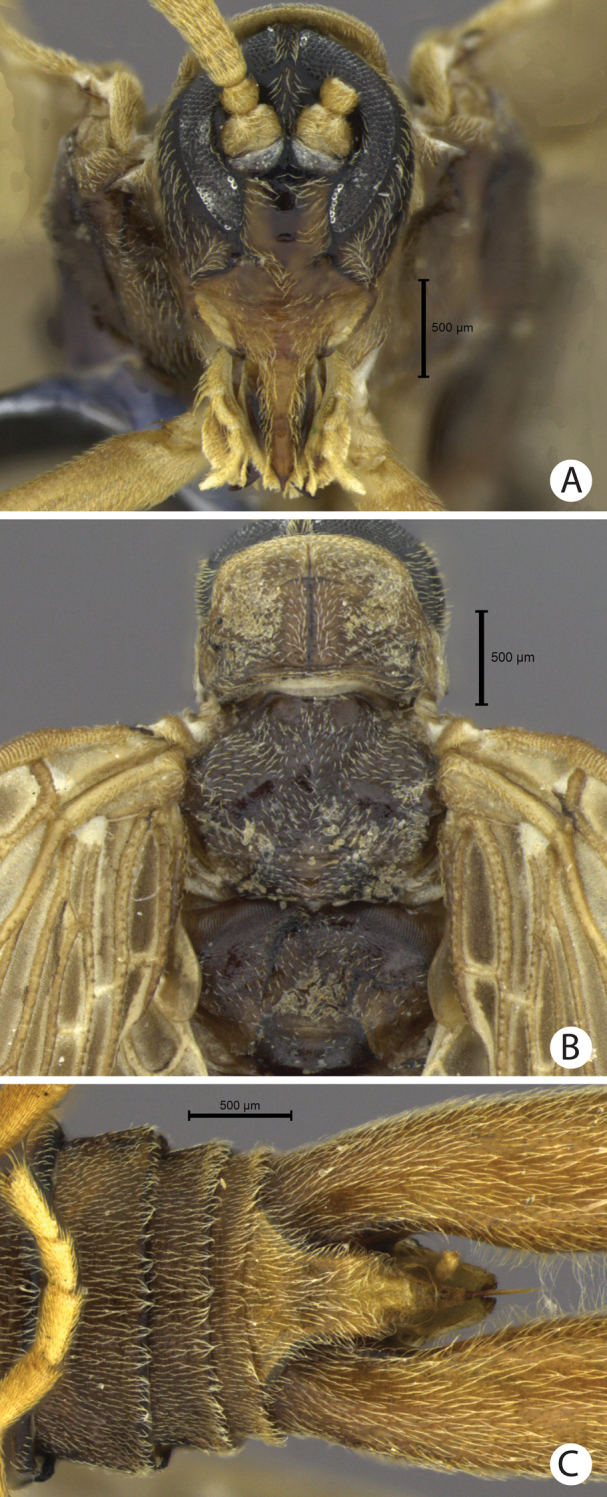
**A–C** *Austromerope brasiliensis* sp. n. **A** Head, frontal view **B** Thorax, dorsal view **C** Abdomen tip, ventral view.

(male holotype). Body length: 20 mm; wing length: 13.8 mm. Head: Eyes black, encircling antennae and almost touching each other dorsally (Fig. 4a); cuticle between and around eyes dark-brown (Fig. 4a). Ocelli absent. Frons, clypeus, labrum, and gena brown. Mandible dark-brown, palps pale (Fig. 4a). Antennae pale, scape broader than pedicel, which has the same width as basal flagellomeres in frontal view; 47 flagellomeres (each wider than long), the basal and apical ones thinner in lateral view (Fig. 3e). Head and body completely covered by small pale setae. Thorax: Pronotum brown, except for 2 black lines, 1 medial longitudinal, the other transverse, sub-apical (Fig. 4b); anterior border pale and folded dorsally. Pronotum as wide as head. Meso- and metanotum dark brown, both broader than pronotum, metanotum with anterolateral region serrated and modified into stridulatory organ (Fig. 4b). Thoracic pleura brown to dark brown. Legs: All pale and of same length (Fig. 2a). Tibia with 2 apical spurs. Tarsi 5 segmented. Pretarsal claws with small teeth (Fig. 3f). Wings: (Fig. 2) Semi-elliptical, membrane fuscous but hyaline around crossveins, slightly darker in inferior area of the wing. Membrane under first branch of M (until the 1st fork) hyaline in hind wing. Veins pale. Costal vein with many transverse rows of small, pale setae. Sc with many branches parallel to C. Rs and M divided into 11 and 9 branches respectively in the forewing, and 12 and 11 in the hind wing. Cu2 ending close to Cu1 in apical half of forewing. Cu2 bifurcated in hindwing. Jugal lobe modified into stridulatory organ in forewing. Abdomen: Segments I-IV slightly darker than others (Fig. 3a) and with sparse pillosity medially. Segments V-IX brown with denser pillosity medially (Fig. 4c) [glabrous in *Merope tuber*, Fig. 8c]. Tergite I longitudinaly divided medially. Tergite IX with posterior margin truncated and longer than tergite VIII (Fig. 3b). Terminalia: Anal dorsal plate curving down in lateral view, apex truncated with acute projection medially in dorsal view (Fig. 3b). Cercus small, rounded, with small pale setae (Fig. 3b). Genital forceps pale, long, slightly longer than abdomen (Fig. 1). Basal segments of forceps subparallel, with proximal region covered by long pale setae and with inner margin expanded (Fig. 3b); distal extremity with small truncate expansion on inner margin (Fig. 3d). Apical segment curved, with apex truncate (Fig. 3d). Basal segment broader and more than 2x longer than apical segment (Fig. 3c).

##### Etymology.

The specific epithet was named for the country where the specimen was collected.

##### Discussion.

**Figure 5.**
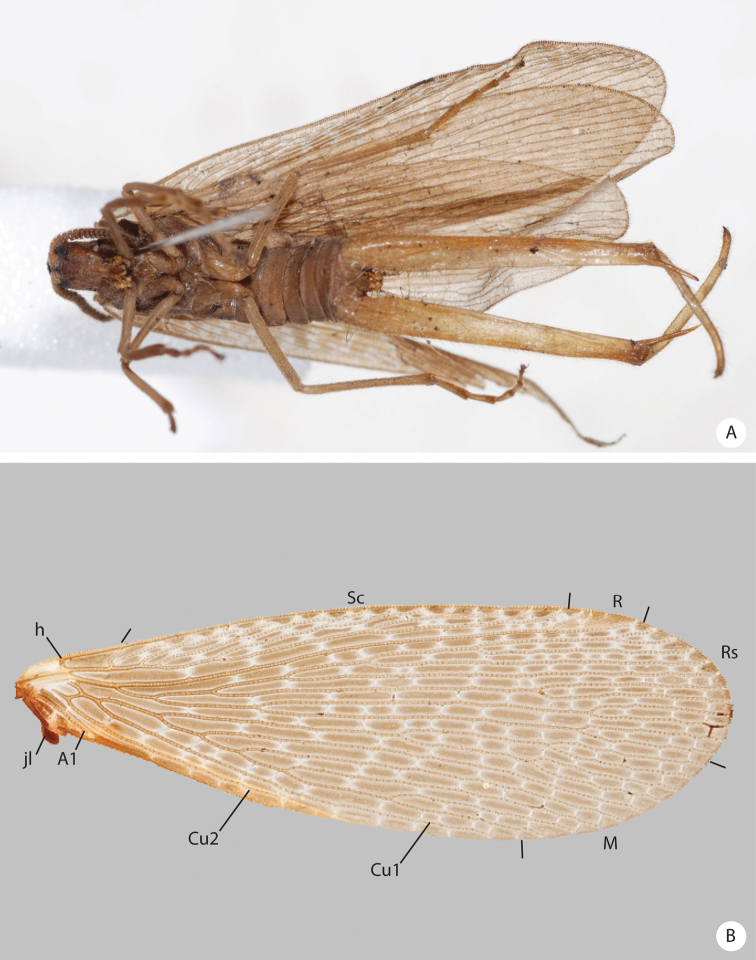
**A–B** *Austromerope poultoni*. **A** Ventral view **B** Forewing. Abbreviations: **A** Anal **Cu** Cubitus **h** humeral **jl** jugal lobe **M** media **R** Radial **Rs** Radial sector **Sc** Subcosta.

Killington (1933) described the genus *Austromerope* from Australia and pointed out several characters that differentiated it from *Merope*, including: prothorax as wide as head (Figs 4b, 8a); pretarsal claws with small teeth (Fig. 3f); apical spine on the basal segment of the forceps (Fig. 5a); jugal lobe of forewing narrow and elongate (Fig. 5b); Rs with 5 or 6 bifurcations (Figs 2b, 5b); M with 2 bifurcations; the great number of crossveins; and costal crossveins parallel to C (Figs 2b, 5b). The new species described here shares all of these defining characters with *Austromerope poultoni* (except the apical spine on the basal segment of the forceps, which is just a truncated expansion in the Brazilian species). In addition, the new species and the Australian one have some other characters in common, such as: the color pattern of the wings and body; antennal flagellomeres wider than long (Fig. 3e); tergite IX longer than tergite VIII (Fig. 3b); the shape of the basal segment of the forceps (subparallel); and the size of the basal segment of the forceps, which is more than 2× longer than the apical segment (Fig. 3c) (almost 1.5 longer in *Merope tuber*, Figs 7a, 8b). Because of all these shared characteristics we have decided to include the Brazilian species in the genus *Austromerope* and not in a new genus, despite the disjunct distribution.

*Austromerope brasiliensis* can be separated from *Austromerope poultoni* by Cu1 not connected with M in *Austromerope brasiliensis* (Fig. 2b), but connected with M by a short distance basally in *Austromerope poultoni* (Fig. 5b); by the truncated apical margin of abdominal tergite IX (Fig. 3b), which is rounded in *Austromerope poultoni* (Killington 1933; Fig. 5); by the truncated apical expansion of the basal segment of the forceps (Fig. 3d), which has a strong apical spine in *Austromerope poultoni* (Fig. 5a); and by the truncated apex of the apical segment of the forceps (Fig. 3d), which is pointed in *Austromerope poultoni* (Fig. 5a).

##### Family distribution.

This is the first record of Meropeidae in the Neotropical region, and together with Bittacidae, it is one of the only two families of Mecoptera existing in Brazil (Machado et al. 2009). The family’s disjunct distribution was discussed by Byers (1973), who compared it to the distribution of the other mecopteran families. He noted that the North American fauna seemed to be more closely related to the Eurasian fauna, with some families such as Boreidae, Panorpodidae, and Panorpidae, occurring only in these areas. He further commented that the Australian fauna, in turn, was basically endemic except for 1 family, Nannochoristidae, which also occurred in South America. Consequently, Byers (1973) suggested that South America was probably the connection between the North American species and the Australian one. Furthermore, he also wondered, in case his hypothesis was true, if there might be another Meropeidae species waiting to be discovered in South American forests. The discovery presented here therefore confirms Byers’ (1973) hypothesis, but likely for a different reason. The subsequent description of the fossil species *Boreomerope antiqua* Novokschonov, 1995 suggests that another distribution hypothesis needs to be considered.

Novokschonov (1995) discussed the relationships among the 3 Meropeidae genera, and highlighted the difficulty to decide which 2 are sister species. Furthermore, he mentioned different characteristics that can be used to approximate any genus, such as Cu1 connected to M for a short distance and Sc branching pattern of Sc, which are shared between *Austromerope* and *Boreomerope*; the low number of Rs and M branches indicating a closer relationship between *Merope* and *Boreomerope*; and the short length of Cu2, shared by *Merope* and *Austromerope*. The discovery of the new *Austromerope* species from Brazil helps rectify some of these inconsistencies indicated by Novokschonov (1995). The character used to join *Merope* and *Austromerope*, the short length of Cu2, is not useful since it is much longer in *Austromerope brasiliensis* than in any other species. The features suggesting a relationship between *Austromerope* and *Boreomerope* are also problematic; the connection between Cu1 and M does not occur in *Austromerope brasiliensis*, and the number, length, and shape of the Sc branches, actually appear more similar between *Merope tuber* and *Boreomerope antiqua*. On the other hand, the large number of branches of Rs and M in both species of *Austromerope* suggests that it is probably a constant feature within the genus, and consequently the low number in *Merope* and *Boreomerope* suggests these 2 genera are closely related. Moreover, the small number of crossveins and the broad area between Sc and R1 may suggest a closer relationship between *Merope* and *Boreomerope*. If the hypothesis of *Merope* + *Boreomerope* is true, it is notable that the species from the same hemisphere are closely related to each other. Therefore, we deduce that after the breakup of Pangea the family was divided into 2 main branches, 1 in the southern hemisphere, represented now by *Austromerope*, and 1 in the northern hemisphere, currently represented by *Merope tuber*. *Boreomerope antiqua* is from the Middle Jurassic, a period when the continents had already split. It would therefore belong to the northern branch. The widespread distribution of Meropeidae corroborates the fact that the family arose when all continents were connected. In fact, the current global distribution of the mecopteran family Bittacidae (Penny 2012) as well as the presence of some Mecoptera fossils from the Permiam period (290-248 MYA), also when all the continents were united (Grimaldi and Engel 2005), further corroborate this hypothesis. The similarities between the 3 extant species of Meropeidae suggest that despite the early bifurcation and the current distribution of the family, the evolution of the group was very conservative, as mentioned by Byers (1973).

##### Conservation.

The most intensely studied and explored area for Mecoptera in Brazil is the Southeast region (Machado et al. 2010), where *Austromerope brasiliensis* was collected. However, despite all previous collecting efforts in this area the species had never been recorded before. The specimen was collected in a private ranch near a forest fragment surrounded by farms in the Atlantic Forest biome, one of the most threatened in Brazil. The discovery of this new relict species is an important signal to reinforce the conservation of this biome. Certainly there are many more mecopterans species yet to be discovered in these forests.

**Figure 6.**
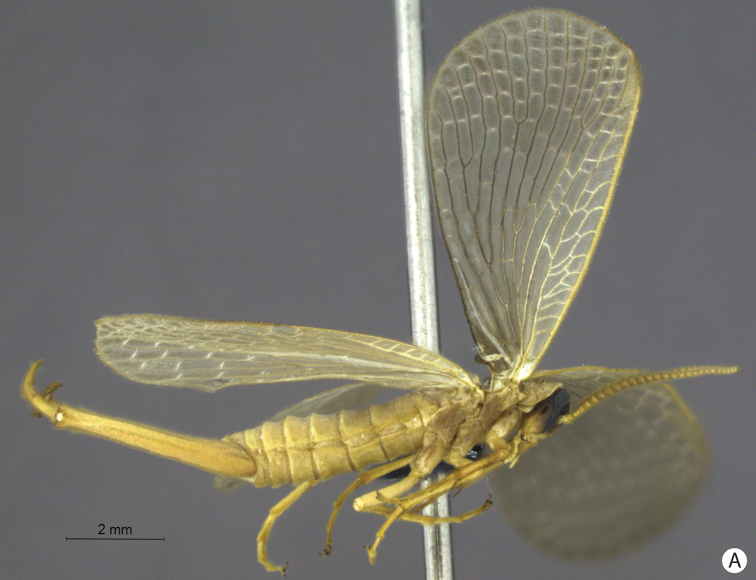
*Merope tuber* lateral view.**.**

**Figure 7.**
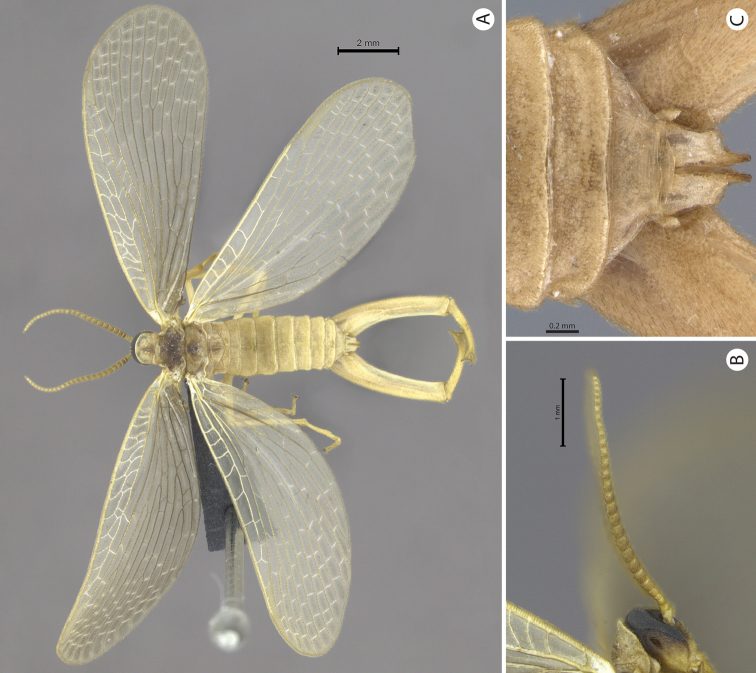
**A–C** *Merope tuber*. **A**. Dorsal view **B** Antennae, lateral view **C** Abdomen tip, dorsal view.

**Figure 8.**
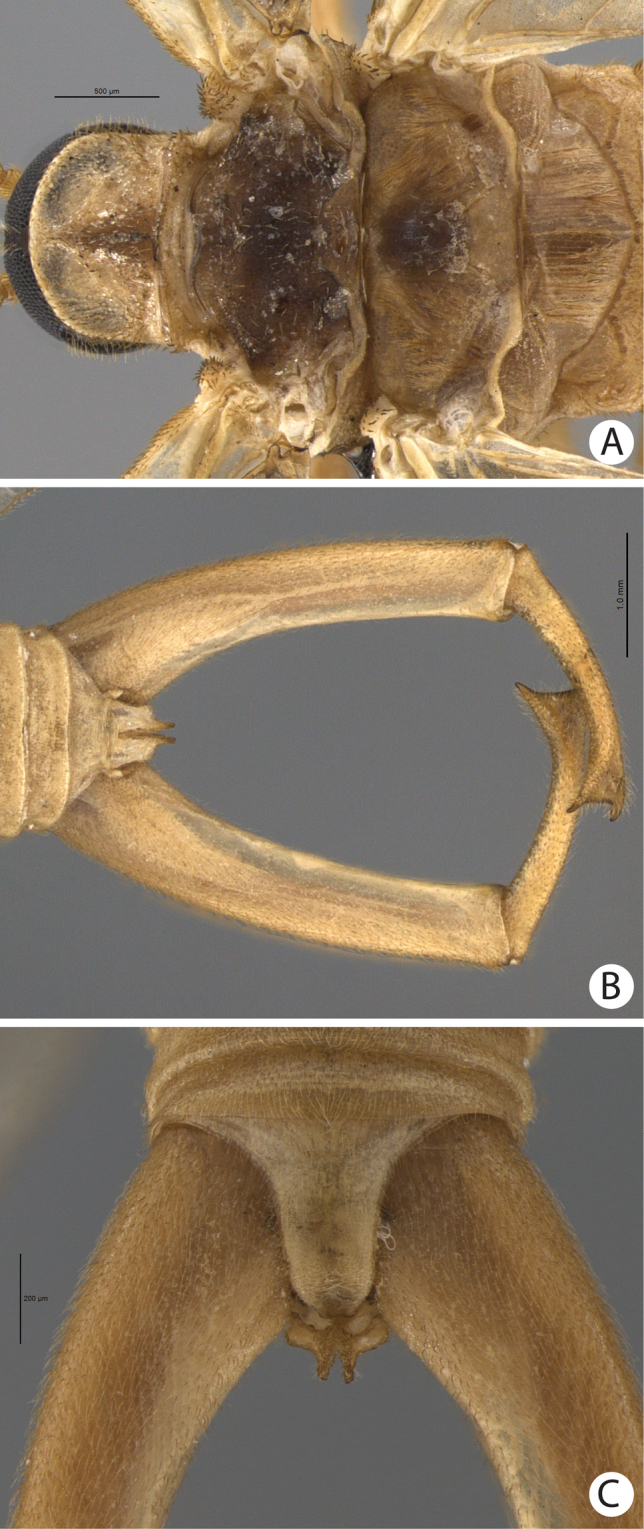
**A–C** *Merope tuber*. **A** Thorax, dorsal view **B** Terminalia, dorsal view **C** Abdomen tip, dorsal view.

**Figure 9.**
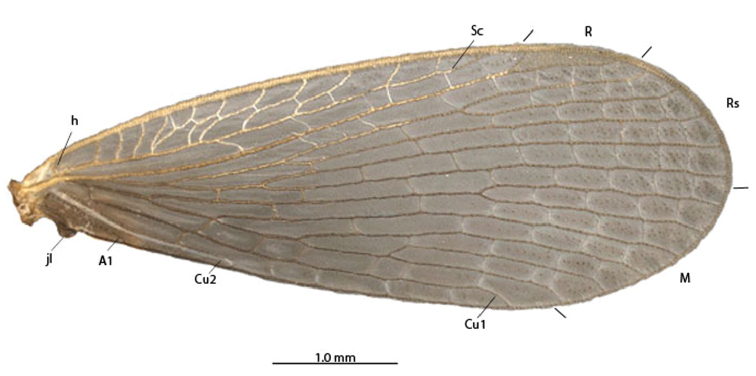
*Merope tuber*, forewing. Abbreviations: **A** Anal **Cu** Cubitus **h** humeral **jl** jugal lobe **M** media **R** Radial **Rs** Radial sector **Sc** S

## Supplementary Material

XML Treatment for Austromerope brasiliensis
